# Acute drug reaction to phenylephrine and tropicamide collyrium in a late-preterm newborn: a case report

**DOI:** 10.1186/s12887-022-03459-z

**Published:** 2022-07-07

**Authors:** Francesco Baldo, Laura Travan

**Affiliations:** 1 Department of Medicine, Surgery and Health Sciences, University of Trieste, Trieste, Italy; 2 Institute for Maternal and Child Health, IRCCS Burlo Garofolo, Trieste, Italy

**Keywords:** Phenylephrine, Collyrium, Bradycardia, Newborn, Case report

## Abstract

**Background:**

Collyrium administration is a common procedure in the neonatal ward, both in preterm and at term babies. Various molecules are used to induce mydriasis and cycloplegia: among them, phenylephrine and tropicamide are the most popular, and their administration is generally considered safe.

**Case presentation:**

A 35 + 2 weeks-old, 2510 g, well-appearing male newborn required an ophthalmologic evaluation after a doubtful red reflex test. A collyrium with 1% phenylephrine and 0.95% tropicamide was administered prior to the consult, one drop per eye. Two minutes after the administration, the baby developed a severe apnea that required tactile stimulation. Moreover, the area around his eyes became visibly pale. Three minutes later, the baby became severely bradycardic (59 bpm), but remained in good general condition, so that resuscitation maneuvers were not required. Bradycardia lasted for almost three hours and then gradually resolved.

**Conclusions:**

Cardiopulmonary manifestations, such as bradycardia and even cardiopulmonary arrest, are severe complications that can happen after phenylephrine collyrium administration in preterm newborns. However, they have been described in babies below 1500 g or with concurrent respiratory manifestations. Our patient, on the other hand, was late preterm, and never required a ventilatory support prior to the collyrium administration. Practitioners who deal with premature babies, even if late preterm, must be aware of these possible complications and administer phenylephrine collyrium carefully, where cardiopulmonary resuscitation equipment and personnel are available.

## Background

Collyrium administration is a common procedure in the neonatal ward. In fact, preterm babies are routinely screened for retinopathy of prematurity, while at term newborns undergo ophthalmological evaluation in case of abnormal red reflex test [1, 2]. Anti-parasympathetic drugs (such as tropicamide and cyclopentolate) and sympathetic agonist (mainly phenylephrine), whether alone or combined, are commonly used to induce mydriasis and cycloplegia. The goal is to perform an ideal eye dilation, allowing peripheral retinal examination without developing side effects. Phenylephrine and tropicamide are the most common molecules used for newborn’s eye drops [3]. As anticholinergic agent, tropicamide may be preferred to cyclopentolate, especially in low weight children, because its average onset time is shorter, and its recovery time is almost five times faster [4].

Phenylephrine and tropicamide collyrium administration is generally considered safe. Altay et al., for example, described a cohort of 60 preterm babies in which mean systolic and diastolic pressure, as well as oxygen saturation, did not change significantly after phenylephrine and tropicamide administration. Food intolerance and vomiting were also not observed after the instillation of eye drops. However, 4 infants that were previously diagnosed with respiratory distress syndrome showed a general condition deterioration 40–70 min after dilated fundus examination [5]. Furthermore, a retrospective study by Lux et al. showed that, in 520 newborns under 1500 g, corresponding to 1033 ocular fundus examinations, no case of death could be ascribed to the use of eye drops [6].

Nowadays, phenylephrine and tropicamide collyrium are available at various concentrations, and there’s a lack of consensus on the ideal dosage to administer to newborns. As a result, mydriatic protocols may differ among hospitals, even in the same country.

## Case presentation

We describe the case of a 35 + 2 weeks-old, 2510 g, well-appearing male newborn, born from vaginal delivery. Apgar was 9 and 10 at 1 and 5 min of life, and skin-to-skin care with his mother was started right after birth. On first visit, a partial syndactyly of the second and third toes of both feet was identified, as well as arachnodactyly and pectus excavatum. In consideration of these anomalies, possibly related to a connective tissue disorder, and due to the presence of a dark red reflex test, an ophthalmologic evaluation was required.

**Fig. 1:**
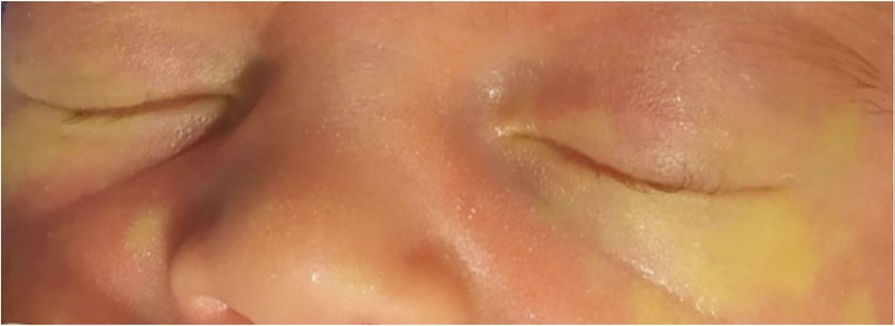
Periocular pallor after collyrium administration

As per our Center protocol, we prepared a mydriatic collyrium by diluting a standard phenylephrine and tropicamide product, reaching concentrations of 10 mg/mL (1%) and 9.5 mg/mL (0.95%) respectively. One drop per eye of this solution should be administered thrice, once every ten minutes. Two minutes after the first administration, the baby developed an apnea that required tactile stimulation, followed by complete normalization of his vitals parameters. Subsequently, the area around his eyes became visibly pale (Fig. 1). Three minutes after the apnea, the boy became severely bradycardic, with a minimum of 59 bpm. Arterial blood pressure and oxygen saturation always remained within the normal range. Body temperature was adequate. As a precautionary measure, high flow nasal cannula was started at 6 L/min. An electrocardiogram confirmed the presence of sinus bradycardia. Blood exams showed no abnormalities. Ultimately, the baby di not required resuscitation maneuvers. Periorbital discoloration resolved within one hour. Bradycardia lasted for almost three hours and then gradually resolved. The patient’s vital parameters were normal for the following two days, after which he was discharged.

## Discussion and conclusions

Overall, phenylephrine and tropicamide collyrium is well tolerated in the neonatal population. However, an exception must be taken for premature babies, especially with a low birth weight (i.e., less than 1500 g) [7]. Topical ophthalmic medications bypass the gastrointestinal and hepatic metabolism and are directly absorbed into the systemic circulation. Consequently, the presence of an immature cardiovascular and nervous systems makes premature infants highly susceptible to the toxicity of these medications. Phenylephrine, when absorbed systemically, can induce a transitory hypertensive and bradycardic response, which can lead to cardiopulmonary arrest (CPA). Tropicamide itself, when combined with phenylephrine, can synergically leads to CPA. Periocular pallor, on the other hand, is a relatively common, local adverse event, caused by overflow and absorption of phenylephrine through the skin.

The potential adverse events of collyrium administration in premature babies have already been described by various authors. For example, in their systematic review of the literature, Kremer et al. described multiple adverse events after the use of mydriatic collyrium in the screening for retinopathy of prematurity, involving the cardiovascular, respiratory, gastrointestinal, and nervous systems [8]. Some of them were severe, such as desaturation, necrotizing enterocolitis (NEC), and seizures, or even life-threating, such as apnea and cardiopulmonary arrest [9]. As for the cardiovascular adverse events, fourteen cases were reported in total, and phenylephrine was administered in all of them. Bradycardia was described in five cases (with pulselessness in one), tachycardia in three, and arterial hypertension in one. No cases of death were reported by the authors.

Mitchell et al. described a cohort of 50 newborns with a mean post-conceptional age of 32.8 weeks [10]. Of the 39 babies who were not on ventilation, nine of them developed apnea within 24 h from the eye examination, and twelve of them within 25 to 48 h. Apnea was therefore significantly associated with collyrium administration, while heart rate differences, cyanosis and seizures were not.

A case of cardiopulmonary arrest after phenylephrine and tropicamide drops administration was described also in a 6-week-old female infant born at 31 weeks of gestation, as reported by Agrawal et al. Resuscitation was promptly initiated with return of spontaneous circulation after 1–2 min [11]. The baby was admitted to the pediatric intensive care unit and monitored overnight. The interesting aspect of this paper lies in the fact that, even if the baby was discharged in the previous days and CPA happened outside the neonatological ward, the resuscitation procedure was started immediately by adequate personnel.

As reported in the case description, our patient never required resuscitation maneuvers. In fact, his initial apnea resolved after tactile stimulation and then, despite being bradycardic, the baby always remained in good clinical conditions. Cardiopulmonary resuscitation is not indicated in newborns with isolated bradycardia, i.e., without oxygen desaturation. The child was closely monitored in the Neonatal Intensive Care Unit until the arrythmia resolved, and always remained in good clinical conditions.

Interestingly, we also like to highlight that our patient’s bradycardia developed before the actual fundus examination, and thus his clinical symptoms are not attributable to the cumulative effect of stress and pain of the ophthalmological examination.

Apart from newborns, adverse reaction to phenylephrine and tropicamide collyrium are very rare. A 2015 systemic review and metanalysis that included 916 participants showed that, in the adult population, 2.5% phenylephrine eye drops did not lead to clinically relevant change in blood pressure and heart rate, and 10% phenylephrine collyrium caused only short-lived blood pressure and heart rate rise [12]. Severe adverse events to phenylephrine collyrium are described mainly in relation to ophthalmological procedures, as reported by Abdelhalim et al. [13]. The authors described the case of a 4-year-old boy who developed severe hypertension and acute pulmonary edema during retinal surgery (i.e., after surgical incision of the eye) due to systemic absorption of topical phenylephrine eye drops. The severe adverse reaction was characterized by an abrupt drop in oxygen saturation, a rapid increase of blood pressure (220/120 mmHg) associated with tachycardia (140 beats per minute) and the development of chest crepitations and intratracheal secretions. Hydralazine and furosemide were administered and, after 20 min from the onset of the event, all vital parameters went back to normal.

Interestingly, our case is different from what is already known in the medical literature. Our patient, in fact, was late-preterm and weighted more than 1500 g, which was an adequate weight for his gestational age. To our knowledge, this is the first case report of a severe adverse event after collyrium administration in this type of newborn. Also, the baby did not have comorbidities, especially from a respiratory point of view, since he never required any ventilatory support, neither invasive nor non-invasive. Furthermore, the collyrium that we routinely administer in our Neonatal Intensive Care Unit is significantly diluted, and both phenylephrine and tropicamide concentrations are within the accepted safe range. Lastly, various methods have been previously described to prevent systemic absorption of ocular drugs in newborns, such as using small-size droppers, reducing the number of drops and the frequency of administration, and blotting the overflowing tears that are mixed with the drugs [14]. All of them are routinely used in our department and were applied in this case as well.

Our case highlights the fact that collyrium administration can cause adverse events also in late preterm babies without respiratory comorbidities. Practitioners who deal with newborns must be aware of the possible severe complications caused by phenylephrine and tropicamide eye drops, and that collyrium should be administered only where cardiopulmonary resuscitation equipment and personnel are rapidly available.

## Data Availability

Not applicable.

## Abbreviations

CPA: Cardiopulmonary arrest
NEC: Necrotizing enterocolitis
L/min: Liters per minute
Bpm: Beats per minute
